# Influence of Irradiance and Wavelength on the Antioxidant Activity and Carotenoids Accumulation in *Muriellopsis* sp. Isolated from the Antofagasta Coastal Desert

**DOI:** 10.3390/molecules27082412

**Published:** 2022-04-08

**Authors:** Daniela Diaz-MacAdoo, Maria Teresa Mata, Carlos Riquelme

**Affiliations:** Centro de Bioinnovación de Antofagasta (CBIA), Facultad de Ciencias del Mar y Recursos Biológicos, Universidad de Antofagasta, Antofagasta 1271155, Chile; maria.mata@uantof.cl (M.T.M.); carlos.riquelme@uantof.cl (C.R.)

**Keywords:** *Muriellopsis* sp., light-emitting diode, wavelength, irradiance, carotenoids, lutein, antioxidant activity

## Abstract

Microalgae are a valuable natural resource for a variety of biocompounds such as carotenoids. The use of different light spectra and irradiance has been considered as a promising option to improve the production of these compounds. The objective of this study was to evaluate the influence of different wavelengths (white, red, and blue) and irradiances (80 and 350 µmol photons/m^2^/s) on the photosynthetic state, total carotenoids and lutein productivity (HPLC), lipids (Nile red method) and antioxidant activity (DPPH) of the microalgae *Muriellopsis* sp. (MCH-35). This microalga, which is a potential source of lutein, was isolated from the coastal desert of Antofagasta, Chile, and adapted to grow in seawater. The results indicate that the culture exposed to high-intensity red light showed the highest biomass yield (2.5 g/L) and lutein productivity (>2.0 mg L^−1^day^−1^). However, blue light was found to have a stimulating effect on the synthesis of lutein and other carotenoids (>0.8% dry wt). Furthermore, a direct relationship between lipid accumulation and high light intensity was evidenced. Finally, the highest antioxidant activity was observed with high-intensity white light, these values have no direct relationship with lutein productivity. Therefore, the findings of this study could be utilized to obtain biocompounds of interest by altering certain culture conditions during the large-scale cultivation of MCH-35.

## 1. Introduction

Microalgae are small unicellular photosynthetic organisms living in saline or freshwater environments and are one of the main components of the trophic chain [1]. The potential of microalgal photosynthesis to produce valuable compounds or for energetic use is widely recognized due to its more efficient utilization of sunlight energy compared with higher plants. They also contain several bioactive compounds that can supplement the nutritional and energy needs of humans, such as proteins, lipids, carbohydrates, vitamins, and antioxidant pigments (e.g., carotenoids). Microalgal biomass production can be highly influenced by light intensity, wavelength, temperature, salinity, pH, and environmental stress; therefore, by altering these culture conditions, the microorganisms could be induced to produce high concentrations of valuable biocompounds [2].

It is well documented that exposure of microalgae to more than one stress at the same time, can contribute to a stronger effect on the productivity of algae and their ability to accumulate lipids or valuable carotenoids [3]. For example, in the green alga *Chromochloris zofingiensis* (ATCC30412) the high light + salt stress had synergistic effects on the astaxanthin and lipid biosynthesis [4]. Other reports found that phosphate starvation and light stress increased astaxanthin productivity in *Haematococcus pluvialis* UTEX 2505 [5]. On the other hand, it has been shown that in the microalgae *Botryococcusonube braunii*, NaCl+ sodium acetate stress increases the lutein content (68.4%) [6]. Knowledge of these factors is key to establishing production systems on a larger scale for the different compounds of interest. Based on bioprocess engineering, it is possible to model the systems where each of the culture parameters are integrated in combination with the different stress factors to establish the best culture conditions in the final system. For example, the reports of Kroumov et al. [7] evaluate how to combine the knowledge of the modeling of subsystems considering the relationships between them, and how these interrelationships influence the general PBR modeling procedure for microalgae culture.

Among the variety of biocompounds produced by microalgae, carotenoids constitute a class of terpenoid pigments derived from a 40-carbon polyene chain [8]. They are widely distributed in nature and responsible for a wide variety of colors [9]. Carotenoids are light-harvesting complexes present in thylakoid membranes, and they are known to protect the photosynthetic apparatus in plants from excess light through energy dissipation and the ability to deactivate ^1^O_2_ [10]. In humans, carotenoids exhibit antioxidant activities that are known to mitigate the harmful effects of free radicals, thereby providing protection from compromised immune response, premature aging, certain cancers, cardiovascular diseases, arthritis, cataract neurodegeneration, and obesity [2,11]. For this reason, the world market value of carotenoids was USD 1.5 billion in 2017 and is projected to reach USD 2.0 billion by 2022 [12]. 

Lutein and zeaxanthin are xanthophyll carotenoids that have gathered increasing attention because of recent studies indicating that they might prevent the effects of age-related macular degeneration (AMD) [13,14]. Among the several microalgae that have been reported as lutein producers, *Muriellopsis* sp. and *Scenedesmus almeriensis* have been tested in growth conditions that could be considered for large-scale biomass production [15,16]. The production of lutein in microalgae varies not only among species but also under different conditions, such as light intensity and media compositions [17]. Light is the major factor influencing algal growth, which has been confirmed through studies of occurring relationships between light intensity and algal density, chlorophyll concentration, and photosynthesis activity [18]. At low intensities, light can become growth-limiting as the mutual shading or self-shading of cells causes steep gradients of light. In contrast, when algae are exposed to excess light intensities, it can result in oversaturation of the electron transport chain capacity resulting in photo-oxidative damage [19]. Photo-acclimatization mainly consists of biochemical and morphological changes in the algal cells for efficient utilization of the incident light in the cultures or for the protection of the photosynthetic machinery from excess light.

Sunlight is a natural source of light and is cost-effective, but it has variations caused by seasonal and weather changes throughout the year and light cycles. Microalgae have evolved specific photoreceptors in order to sense and respond to changes in light quality through phototactic or metabolic responses [20]. The utilization of light depends on their pigment composition (mainly chlorophylls, carotenoids and phycobilins). These absorb light in different spectral regions (chlorophyll λ = 400–550 nm and 630–675 nm, carotenoids λ = 400–550 nm and phycobiliproteins λ = 500–650 nm) [21]. In addition, β-carotene, lutein or fucoxanthin stabilize the photosystems and function to harvest light by transferring excitation energy to chlorophyll. On the other hand, zeaxanthin or astaxanthin can provide photoprotection of reactive oxygen species (ROS) [20,22].

Due to this, artificial illumination, such as fluorescent lamps and light-emitting diode (LED) lights are increasingly being used in microalgae culture [23]. Several studies utilizing artificial illumination for the cultivation of microalgae have been carried out, focusing on obtaining a higher yield and productivity of bioactive compounds of interest. 

Based on these antecedents and due to the unique conditions of the northern Chilean desert, such as its high luminosity, and understanding that light is a key factor in the production of compounds of interest, this research was carried out on a green microalgae *Muriellopsis* sp. (MCH-35), isolated from the coastal desert of Antofagasta and adapted to grow in seawater [24,25]. We have focused on investigating the effects of different light spectra (red, blue, and white light) and light intensities (80 and 350 µmol photons/m^2^/s) in the culture on the growth, biomass production, photosynthetic activity, accumulation of carotenoids (including lutein), lipids and antioxidant activity of microalgae extracts. These results could provide relevant approaches (altering culture conditions such as wavelength and irradiation) to produce valuable biocompounds by large-scale cultivation of MCH-35.

## 2. Results and Discussion

### 2.1. Comparison of the Effect of Irradiance and Wavelength on the Growth Kinetics and Biomass Production of the MCH-35 Microalgae

**Figure 1:**
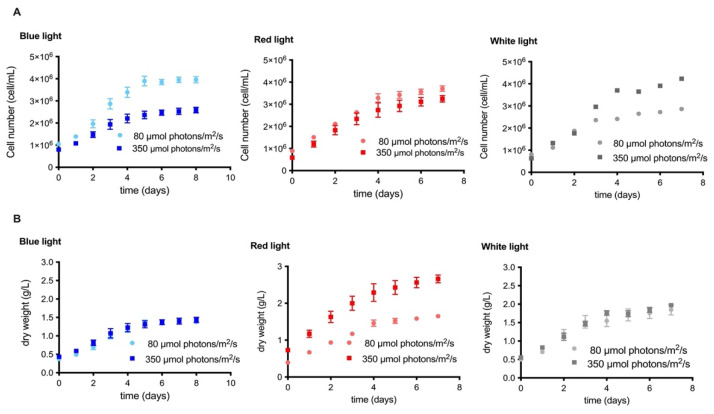
Kinetics of the irradiance effect for each experiment with blue, red, and white light in the MCH-35 microalgae grown at 80 µmol photons/m^2^/s and 350 µmol photons/m^2^/s. (**A**) Cell growth curve (cells/mL) and (**B**) dry weight curve (g/L). The data represents the average of four independent measurements. Data are expressed as the mean ± SD (*n* = 4).

According to our results, the culture with white light at 350 μmol photons/m^2^/s showed a clear increase in cell number with respect to the lower irradiance condition, reaching values ≥4 × 10^6^ cells/mL (Figure 1A). Similar results were observed in other microalgae, such as *Coccomyxa onubensis* [19], which exhibited a direct correlation between the growth rate and irradiance; when cultivated at different intensities of light (50, 140, and 400 μmol photons/m^2^/s), it obtained the maximum growth at 400 µmol photons/m^2^/s. In contrast to white light, under blue and red-light conditions, the microalgae showed a higher growth rate when cultured at 80 μmol photons/m^2^/s. This was clearly evidenced in the culture with blue light, where cell growth values close to 4 × 10^6^ cells/mL (Figure 1A). Other investigations with microalgae *Tetraselmis* sp. and *Nannochloropsis* sp. reported by Teo et al. [26], showed similar results where their growth was analyzed under blue, red, red-blue LED and white fluorescent light; the results showed a better growth curve under the blue wavelength.

Regarding biomass, higher growth was observed in cultures with red light, followed by blue and white light, respectively. No significant differences in biomass were found between irradiances when grown under blue and white light. However, the red-light culture showed an evident increase in dry biomass when the irradiance was at 350 µmol photons/m^2^/s, reaching values close to 2.5 g/L (Figure 1B). These data allow us to infer that the increase in biomass may be due to the increase in cell size when the microalgae were cultivated in this condition.

However, the findings of similar studies, with respect to the effect of light intensity and wavelength on the microalgal growth rate and biomass, vary. For example, in microalgae *Desmodesmus* sp. F-51., Xie et al. [27] demonstrated that biomass increases with increasing irradiance (150 and 750 μmol photons/m^2^/s). Other studies have indicated that blue LEDs can be utilized as an energy source in different microalgae cultures, as it photostimulates the production of biomass and metabolites [26]. This is observed in microalgae *Chlorella fusca* LEB 111 [28], which increases its concentration and biomass productivity with increased intensity of light.

In *Dunaliella salina*, the best growth rate and biomass yield with red light is reported at 128 µmol photons/m^2^/s; at higher values, a decrease in these parameters is detected [29]. On the other hand, in strain *Scenedesmus obliquus* FSP-3, Ho et al. [30], indicated that the wavelength affects biomass production differently; better performance was obtained in one strain with white light and in the other with red light.

This variation in the effects of irradiance and wavelength on microalgal growth, suggests that the influence of wavelength on biomass production and microalgal growth is species-dependent. This is because of differences in light absorption and conversion efficiency among different species [31,32,33,34].

### 2.2. The Effect of Varying Wavelengths and Light Intensities on the Photosynthetic Activity of the MCH-35 Microalgae

It is well known that when microalgae are exposed to high light intensity, they exhibit increased oxidative stress, decreased key proteins of the reaction centers, and decreased rates of photosynthesis and respiration, as well as decreased photochemical efficiency which is accompanied by damage to the photosystem II (PSII) [35].

The observed changes in the maximum PSII quantum yield in samples adapted to light is an indicator used for the measurement of the physiological condition of an organism and represents the effectiveness of the PSII open reaction centers in capturing excitation energy [36].

In previous studies with the microalgae *Nannochloropsis* sp., Das et al. [32] reported that short-wavelength photons, such as blue light, have a high probability of hitting the light-harvesting complex (LHC), resulting in higher photosynthetic efficiency. This is confirmed by the fact that when phototrophic and mixotrophic cultures are exposed to monochromatic blue light, the maximum μmax value is observed. Whereas the minimum μmax value is observed upon exposure to red light with a longer wavelength.

**Figure 2:**
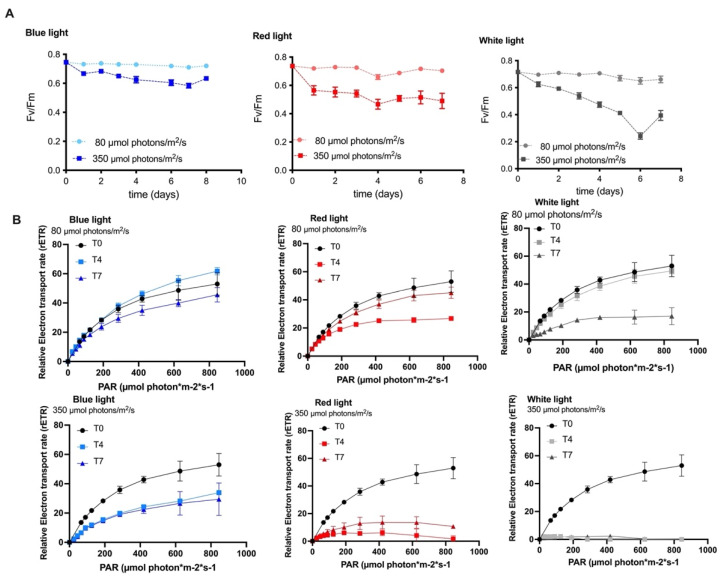
(**A**) Effective quantum yields of PSII [Y (II)] of the MCH-35 microalgae grown under different light wavelengths (red, blue, and white) and irradiances (80 µmol photons/m^2^/s and 350 µmol photons/m^2^/s). (**B**) Relative electron transport rates (ETR) observed on days 0, 4, and 7 of the MCH-35 microalgae grown under different light wavelengths (red, blue, and white) and irradiances (80 µmol photons/m^2^/s and 350 µmol photons/m^2^/s). Data are expressed as the mean ± SD (*n* = 4).

In this study, the maximum quantum yield was observed with all the wavelength conditions; values higher than 0.6 were observed during the whole cultivation process, which was carried out at low irradiance. When the irradiance was increased to 350 µmol photons/m^2^/s, the Fv/Fm values were slightly affected in both blue and red-light conditions; this indicates that the microalgae are tolerant to these culture conditions. On the contrary, the culture under white-light conditions showed a drastic decrease in these values until day six and a slight recovery on day seven. This may be caused by damage to the photosynthetic apparatus in the long term or a low regulation of PSII in response to physiological stress (Figure 2A).

The fast light curves represent a minimally invasive assessment of the effect of light on PSII activity, providing information on the saturation characteristics of the electron transport chain, the general photosynthetic performance, and the photoacclimation of photosynthetic organisms [37,38]. The ETR values were analyzed as an indicator of the productivity of the MCH-35 microalgae exposed to different culture conditions (i.e., varying wavelength and irradiance). Results indicate that when the microalgae were exposed to wavelengths of white, red, and blue light at 80 µmol photons/m^2^/s, the ETR curves on days 0 and 4 remain high without any variations. On day 7, the ETR values decreased considerably in the wavelengths of red and white light. However, under blue light conditions, this significant decrease was not observed, correlating these results with those of Fv/Fm. In the white-light cultures, a drastic decrease in ETR values was observed with high-intensity light. A similar decrease was observed with the red light on day 4; however, on day 7, a slight increase in ETR values, close to 20 µmol electrons/m^2^/s, was observed (Figure 2B). Our results indicate that blue light does not damage the photosynthetic apparatus of the microalgae even when it is cultured at high irradiance, reflected in the Fv/Fm values (Figure 2A) and the ETR curves (Figure 2B). However, high irradiance and white light significantly affect the photosynthetic apparatus and can cause oxidative damage in the microalgae.

### 2.3. Identification and Quantification of Carotenoid Pigments Produced by the MCH-35 Microalgae Exposed to Varying Light Intensities and Wavelengths

**Figure 3:**
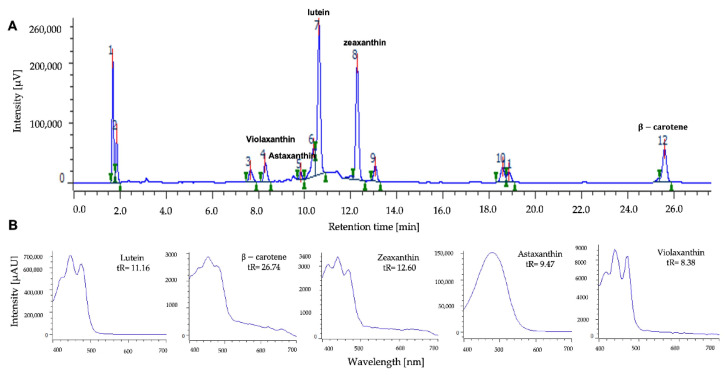
(**A**) Chromatogram representative of microalgal of MCH-35 analyzed using the HPLC method. (**B**) Absorption spectrum in UV-Vis and retention times of the carotenoids violaxanthin, astaxanthin, lutein, zeaxanthin and β-carotene obtained from microalgal extracts by diode array detector.

The carotenoid pigments produced by the MCH-35 microalgae exposed to different culture conditions were analyzed using the HPLC method, and the results indicate the presence of violaxanthin (tR: 8.29), astaxanthin (tR: 9.49), lutein (tR: 10.6), zeaxanthin (tR: 12.27), and β-carotene (tR: 26.74) (Figure 3). These pigments were also described in the study conducted by Schagerl et al. [39] on Chlorophyceae, wherein the presence of neoxanthin, violaxanthin, lutein, zeaxanthin, and β-carotene are reported. 

**Figure 4:**
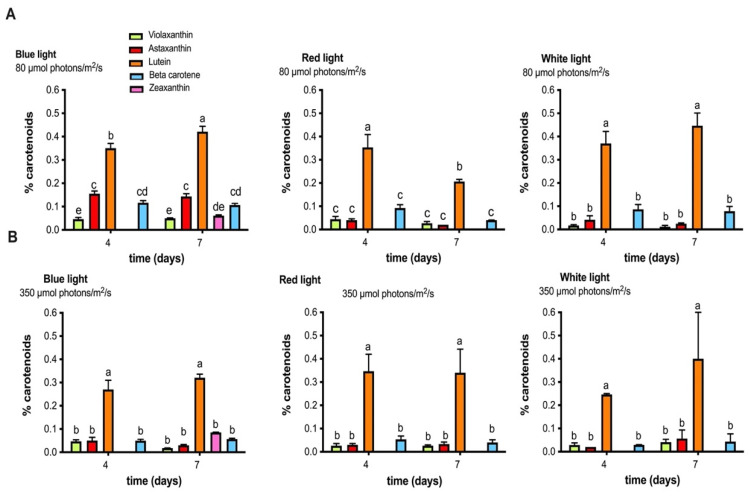
The content of carotenoids (% dry wt), such as lutein, zeaxanthin, violaxanthin, astaxanthin, and beta-carotene, obtained on days 4 and 7 in the microalgae extract of the MCH-35. (**A**) Carotenoid content (%) obtained from blue, red, and white-light culture grown under irradiance of 80 µmol photons/m^2^/s. (**B**) Carotenoid content (%) obtained from blue, red, and white-light cultures grown at an irradiance of 350 µmol photons/m^2^/s. Each bar represents the average of four independent measurements. Statistically different values (*p* = 0.05, ANOVA two-way, with Tukey posthoc test, *n* = 3) are indicated by a different letter for each spectral band.

The analysis of the concentrations of these carotenoids revealed a high content of lutein (between 0.4% and 0.5% dry wt) with respect to the other pigments, presenting this tendency in all culture conditions. In addition, the blue light condition (80 μmol photons/m^2^/s) also stimulated the synthesis of other carotenoids such as astaxanthin, beta-carotene, zeaxanthin, and violaxanthin (Figure 4). On the other hand, it was observed that with red light the lutein content decreases on day 7 compared to the rest of the situations where the content is maintained (Figure 4).

In other reports, Zhao et al. [40] in the marine microalgae *Chlamydomonas* sp. JSC4 demonstrated that the optimal lutein content was obtained under blue light, while the highest production of this carotenoid was observed when the microalgae were exposed to a combination of white and blue light (3:1 proportion) and irradiance of 250 μmol photons/m^2^/s. Similar studies in the acid-tolerant microalgae *C. onubensis* [19], indicated that the amount of lutein produced per cell is inversely proportional to the photon flux density, i.e., lutein productivity increases when the light intensity is low. Pereira et al. [41] reported that illumination of the microalgae *H. lacustris* (formely *H. pluvialis)* with red monochromatic light was optimal to produce green cells, while illumination with red and blue light resulted in maximum accumulation of astaxanthin. Finally, Xu et al. [42] investigated the effect of high-intensity blue, red, and white light on *Dunaliella salina CCAP 19/41*, where growth in red light was associated with the accumulation of carotenoids. Our findings, regarding irradiance, were similar to that of Vaquero et al. [19] and Solovchenko et al. [43], who referred to the fact that the amount of primary xanthophylls in microalgae tended to decrease with high light intensities (blue and white light).

**Figure 5:**
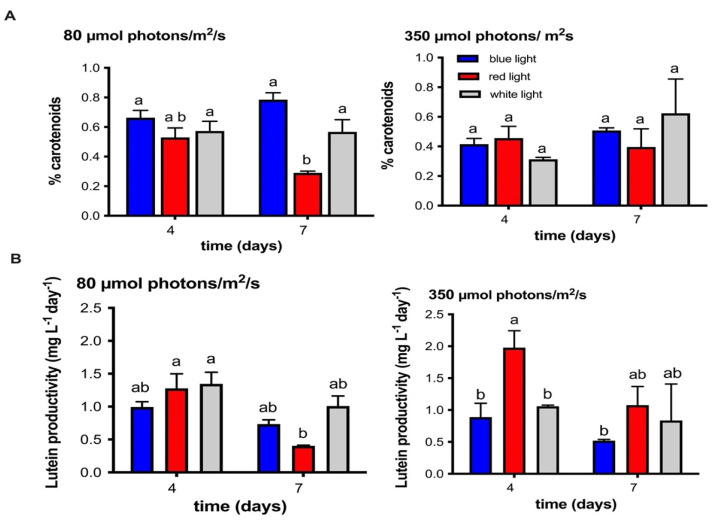
(**A**) Total content (% dry wt) of lutein, zeaxanthin, violaxanthin, astaxanthin, and beta-carotene obtained on days 4 and 7 in the microalgae extract of the MCH-35 grown under white, blue, and red light at irradiances of 80 and 350 µmol photons/m^2^*/*s. Each bar represents the average of four independent measurements. (**B**) Lutein productivity (mg L^−1^ day^−1^) on days 4 and 7 of the MCH-35 microalgae grown under different light wavelengths (red, blue, and white) and irradiances of 80 and 350 µmol photons/m^2^*/*s. Each bar represents the average of four independent measurements. Statistically different values (*p* = 0.05, ANOVA two-way, with Tukey posthoc test, *n* = 3) are indicated by a different letter for each irradiance condition.

With respect to total carotenoids production, our results indicate that MCH-35 cultivation under blue light at 80 μmol photons/m^2^/s presents a higher content of carotenoids, obtaining ≥0.8% dry weight on day 7 of growth versus ≥0.5% dry weight in the high irradiance treatment. Furthermore, red light at 80 μmol photons/m^2^/s negatively affects carotenoids content, showing a drop in concentration on day 7. On the other hand, in the red-light culture high irradiance did not affect the synthesis of pigments, resulting in similar levels of production even after seven days of growth (Figure 5A). Similar studies indicate that different microalgae produce varying concentrations of carotenoids in response to light stress, showing different sensitivity to certain irradiances and wavelengths. For example, in *Dunaliella salina* CCAP 19/30, Xu et al. [44] demonstrated that the increase in light intensity decreases the cellular content of total carotenoids, suggesting that the synthesis of these pigments cannot be the main operating mechanism to protect cells from intense light. In the case of *Dunaliella tertiolecta* [45], a high pigment content was observed under low irradiance conditions. On the other hand, the study of Zhong et al. [46], concluded that blue LEDs could increase the rate of maximum specific growth and photosynthetic pigments in green microalgae *Chlorella vulgaris*, *Auxenochlorella pyrenoidosa* (formerly *Chlorella pyrenoidosa*) and *S. quadricauda* and *Tetradesmus obliquus* (formerly *S. obliquus*).

In terms of lutein productivity, the culture exposed to red light and high irradiance presents the highest productivity values compared to other conditions (>2.0 mg L^−1^day^−1^) (Figure 5B). This is because this condition presented an increase in biomass values, reaching up to 2.5 g/L (Figure 1B). Similar results were exposed by Ma et al. [47] in *Chlamydomonas* sp. JSC4 where the high productivity of lutein was related to the high light irradiation (625 µmol photons/m^2^/s). These results allow us to propose a two-stage cultivation strategy; the first stage would stimulate biomass production with red light at higher irradiance, and in the second stage, induction of lutein and other carotenoids would occur with blue light or white light at lower irradiance. The two-stage cultures have also been proposed in other microalgae to improve the production of pigments of interest. For example, during the growth of the microalgae *H. lacustris*, Xi et al. [48] replaced the traditional light sources with LEDs in two stages. In the first stage, they used red LEDs to stimulate microalgal growth, and later they applied blue LEDs to induce astaxanthin synthesis. In *Dunaliella salina* CCAP 19/18, Han et al. [49] improved the photosynthetic productivity of beta-carotene through a blue-red LED wavelength-shifting system.

### 2.4. Influence of Different Wavelengths and Irradiances on the Lipid Content

Previous research has shown that lighting microalgal cultures with specific wavelengths or a mix of them could not only increase microalgal growth but also stimulate the production of high-value products [50,51]. This is because the composition of biomass, such as lipids, proteins, and carbohydrates, is affected by growth conditions and environments, such as light intensity, temperature, and pH [52].

**Figure 6:**
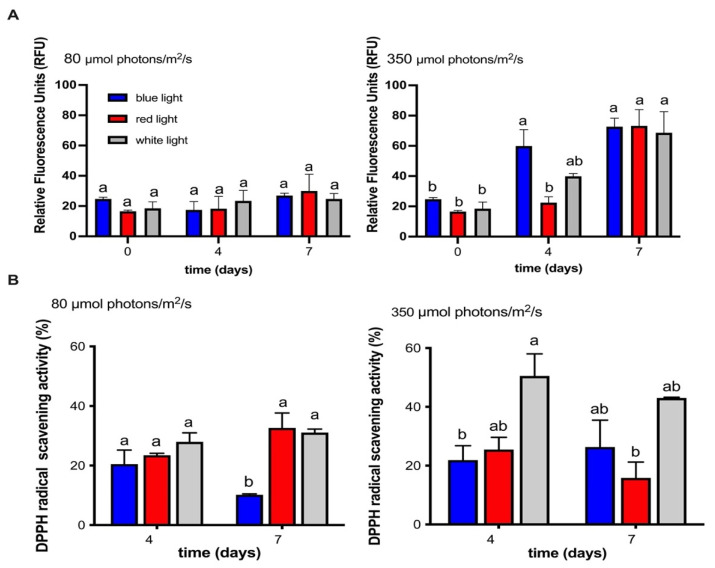
(**A**) Relative Fluorescence Units (RFU) of the MCH-35 microalgae on days 0, 4, and 7 cultivated under irradiances of 80 and 350 µmol photons/m^2^/s and different light wavelengths (red, blue, and white). (**B**) Percentage of DPPH inhibition with the microalgal extract (10 mg/mL) of MCH-35 cultivated under irradiances of 80 and 350 µmol photons/m^2^/s and different light wavelengths (red, blue, and white). Each bar represents the average of four independent measurements. Statistically different values (*p* = 0.05, ANOVA two way, with Tukey posthoc test, *n* = 3) are indicated by a different letter for each irradiance condition.

To determine the influence of wavelength and irradiance on the lipid content in the MCH-35 microalgae, the results of the Nile red technique were analyzed, where high levels of fluorescence (RFU) indicate a higher lipid content. The MCH-35 presents low fluorescence values when cultured at 80 µmol photons/m^2^/s with three different wavelengths (white, red, and blue light) (Figure 6A). However, when cultured at 350 µmol photons/m^2^/s, an increase in fluorescence values was observed in the culture exposed to blue and white LEDs on day 4, reaching a doubling of relative fluorescence values. In addition, for day 7 with red light, an increase in RFU was also observed, reaching the same levels as blue and white light.

In other microalgae, such as *Chlorella vulgaris*, Zhang et al. [53] studied the effect of blue, white, and red light using LED lamps. Their results show a maximum lipid content with blue light, followed by red and white light. *Nannocholorpsis* sp. MUR 266 significantly increases its lipid content with blue light when compared to other light conditions [54]. Finally, in the microalgae, *C. vulgaris*, the increase in irradiance (130 and 520 µmol photons/m^2^/s) with white and red LEDs significantly increased (7.9% and 22.2%, respectively) the lipid content [55]. 

The excess energy produced by the high intensity of light favors the overproduction of lipids; this may be because lipids, such as triacylglycerol, serve as highly concentrated stores of metabolic energy. This requires excess ATP and NADPH, which correspond to products of the photosynthesis process. In this way, excess energy is utilized, which would protect the cells from photochemical damage [56,57].

### 2.5. Influence of Different Wavelengths and Irradiances on the Antioxidant Activity of the MCH-35 Microalgae

Our results showed that the microalgae exhibited antioxidant activity in all the conditions studied. In addition, the culture at 80 µmol photons/m^2^/s did not show differences between light treatments, except for blue light on day 7, where antioxidant activity decreased (Figure 6A). On the other hand, the highest activity was observed at 350 µmol photons/m^2^/s in general, showing that the only condition that maintains high activity over time is white light. (Figure 6B). These values do not show a direct relationship with the carotenoids analyzed such as lutein, zeanxanthin, beta-carotene, violaxanthin and astaxanthin, where a greater accumulation of these was observed at 80 µmol photons/m^2^/s. Similarly, the antioxidant activity did not show a correlation with the higher productivity of lutein obtained from the culture with red light at 350 µmol photons/m^2^/s. These antecedents allow us to deduce that the greater antioxidant activity could be related to the content of other carotenoids or to the synthesis of other compounds that have not been analyzed in this study. Among them, non-enzymatic compounds such as chlorophyll a, chlorophyll b, tocopherols, phycocyanins, phenols, etc. and/or enzymatic antioxidants, such as catalase, superoxide dismutase, glutathione reductase and ascorbate peroxidase. These results agree with data recently published by Cruz-Balladares et al. [25] in which they conclude that there is no direct relationship between antioxidant activity and lutein productivity.

## 3. Materials and Methods

### 3.1. Cell Cultures and Treatment Conditions

**Figure 7:**
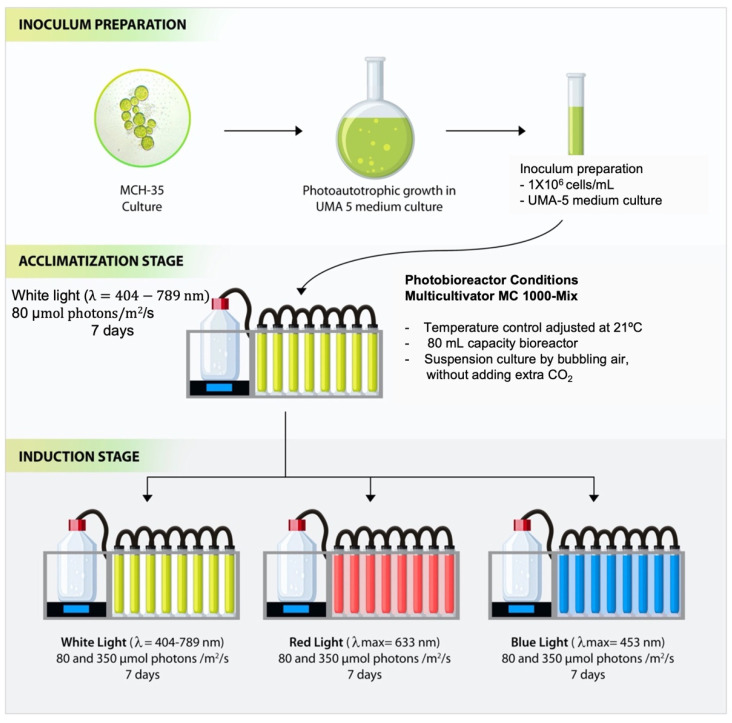
Descriptive two-stage model for the culture conditions of the MCH-35 microalgae grown under different light wavelengths (red, blue, and white) and irradiances (80 µmol photons/m^2^/s and 350 µmol photons/m^2^/s).

Cells were grown photoautotrophically in UMA 5 medium (f/2 modified medium enriched with NaNO_3_ and NaH_2_PO_3_) (NaNO_3_ 0.4 g/L, NaH_2_PO_4_ 0.034 g/L, NaHCO_3_ 0.168 g/L, Zn 0.080 μmol/L, Mn 0.9 μmol/L, Mo 0.03 μmol/L, Co 0.05 μmol/L, Fe 11.7 μmol/L) [58], prepared with filtered seawater (0.2 µm). The suspension culture was constantly mixed and aerated by bubbling air, without adding extra CO_2_. Subsequently, an inoculum of 1 × 10^6^ cells/mL was used in each experiment, and the cells were grown in a Multi-Cultivator MC 1000-Mix equipment (Photon System Instruments, Brno, Czech Republic) at a temperature of 21 °C according to Balladares et al. [25] and Marticorena et al. [24] and the pH of the medium varied between 7.1 and 8.3. The experiments were carried out in 100 mL capacity photobioreactors under blue (λ_max_ = 453 nm), red (λ_max_ = 633 nm), and white (λ = 404–789 nm) LEDs and irradiance intensities of 80 µmol photons/m^2^/s and 350 µmol photons/m^2^/s. These irradiances were selected because 80 µmol photons/m^2^/s corresponds to the acclimatization irradiance of our culture room and 350 µmol photons/m^2^/s corresponds to the maximum irradiance provided by the Multi-Cultivator equipment. Furthermore, both light intensities have been reported by Maltsev et al., [3] as optimal irradiances for the growth of different species of microalgae. The experiment consisted of two stages: the acclimatization stage with white light at 80 µmol photons/m^2^/s for 7 days and the induction stage with six different culture conditions, white light at 80 and 350 µmol photons/m^2^/s, blue light at 80 and 350 µmol photons/m^2^/s, and red light at 80 and 350 µmol photons/m^2^/s for 7 days. Each experiment was performed in four replicates using four independent columns (Figure 7).

### 3.2. Determination of Cell Growth and Dry Weight of the MCH-35 Microalgae

To determine the cell number and dry weight, calibration curves were constructed that related each parameter to its absorbance. The cell number was determined through the Neubauer chamber, and the OD was measured at 680 nm. For the determination of dry weight, 2–5 mL aliquots of the culture were taken (which were previously standardized for species under study) and centrifuged at 8000 rpm; the pellets were washed three times with distilled water to remove excess salts. Subsequently, the samples were dried at 70 °C for 24 h, and their weight was annotated. 

### 3.3. Influence of Light Color and Irradiance on the Photosynthetic State of the MCH-35 Microalgae

Photosynthetic performance estimated by in vivo chlorophyll a fluorescence was determined using the JUNIOR-PAM Chlorophyll Fluorometer (Walz, Effeltrich, Germany). For measurements of the maximum quantum yield (Fv/Fm) and relative electron transport rate (ETR), 1 mL aliquots from each bioreactor were collected in amber Eppendorf tubes. Samples from each culture were adapted to darkness for 10 min.

### 3.4. Organic Carotenoids Extraction from the Microalgae MCH-35

For the extraction of carotenoids, 15 mL aliquots of culture were collected, the samples were washed with distilled water to remove excess salts and centrifuged at 3500 rpm for 10 min, the supernatant was discarded, and the pellet was lyophilized. Subsequently, 10 mg of alumina was weighed in a 1:1 ratio with the microalgal biomass, and 1 mm glass beads were added to the tube along with 1 mL of a three-component mixture (77% ethanol, 17% hexane, 6% H_2_O, and 0.1% KOH); then, gaseous nitrogen was added to each tube to create an inert atmosphere, and the tubes were placed in the bead beater for 2 min pausing in between for 30 s. The mixture was centrifuged at 4500 rpm for 3 min; the organic fraction was collected, and the samples were stored in cold and dark conditions until use.

### 3.5. Determination of the Carotenoids Content in the Microalgae MCH-35

For the determination of carotenoid content, such as lutein, zeaxanthin, violaxanthin, astaxanthin and β-carotene, calibration curves were made for each standard solution with concentrations of 25, 50, 75, 100, 150 and 200 μg/mL. Standard solutions and microalgal extracts were analyzed using the HPLC (LC-4000, Jasco. Tokyo, Japan), equipped with a quaternary pump (PU-2089 s Plus, Jasco, Tokyo, Japan), a diode array detector (MD-4010, Jasco. Tokyo, Japan) and ChromNAV Control Center V.2 software (Jasco. Tokyo, Japan). A reverse-phase column C-18 LiChrospher RP-18 (5 µm) (4:6 × 150 mm). The gradient program used was reported by Cerón-Garcia et al. [59]. The mobile phase corresponds to a mixture of water/methanol (2:8, *v*/*v*) (solvent A) and acetone/methanol (1:1, *v*/*v*) (solvent B). The carotenoids were identified by comparing retention time (tR), calibration curve and spectral characteristics in UV.Vis of zeaxanthin, astaxanthin, lutein, β-carotene, and violaxanthin analytical standards (Sigma-Aldrich, St Louis, MO, USA).

### 3.6. Determination of Total Lipid Production by MCH-35

For total lipid analysis, an inoculum of 2 × 10^5^ cells/mL was made in 300 µL of 25% DMSO and 1 μg/mL Nile red in a 96-well plate [60]. The plate was incubated at 40 °C for 5 min. The analysis was carried out using a fluorometer (GloMax-Multi Detection System, Promega, Madison, WI, USA) at an excitation wavelength of 450 nm and an emission wavelength of 585 nm. The colorimetric blank used was 200,000 cells/mL in 25% DMSO, and the fluorescence intensity was measured in relative fluorescence units (RFU).

### 3.7. Determination of the Antioxidant Capacity of the Microalgae MCH-35 with 2,2-Diphenyl-picrylhydrazyl (DPPH) Radical Scavenging Method

For the analysis of the antioxidant capacity with the DPPH radical reduction method, 150 µL of DPPH 0.2 mM was mixed with 100 µL of microalgal extract in a 96-well plate. The absorbance of the reaction mixture was measured at 517 nm after a 30 min incubation in the dark [25]. The scavenging activity was calculated by the following Formula (1):

(1) $$A_{DPPH}\left(\%\right)=\frac{A_{CONTROL}-A_{SAMPLE}}{A_{CONTROL}}\times 100\%$$

## 4. Conclusions

Our results demonstrate that biomass production, photosynthetic activity, carotenoids (including lutein) synthesis, lipid and antioxidant activity of MCH-35 microalgae are affected by irradiance and wavelength. In addition, the presence of lutein (as the main pigment), astaxanthin, zeaxanthin, violaxanthin and beta-carotene could be evidenced in the microalgal extract. The content analyses of these carotenoids (% dry w.t) showed that the cultivation of blue light at 80 µmol photons/m^2^/s mainly stimulates the synthesis of these compounds. However, the highest productivity of lutein was obtained in the red-light culture at 350 µmol photons/m^2^/s because it is this condition that showed an increase in biomass values (close to 2.5 g/L). Regarding the accumulation of lipids, a relationship with the increase in irradiance was evidenced. Finally, the highest antioxidant activity was observed at 350 µmol photons/m^2^/s where white light maintains high activity at 350 µmol photons/m^2^/s, which did not correlate with the productivity of lutein, this could be related to other carotenoids or other compounds of an antioxidant nature present in the microalgal extract. Therefore, this study provides us with relevant approaches to produce valuable biocompounds in microalgae that are based on the implementation of induction systems, such as by selecting certain spectral bands.

## Data Availability

The data presented in this study are available on request from the corresponding author.

## Sample Availability

Samples of the compounds are not available from the authors.
